# The role of mitochondria in pharmacological ascorbate-induced toxicity

**DOI:** 10.1038/s41598-022-27185-9

**Published:** 2022-12-29

**Authors:** Juan Du, Amanda N. Pope, Brianne R. O’Leary, Brett A. Wagner, Prabhat C. Goswami, Garry R. Buettner, Joseph J. Cullen

**Affiliations:** 1 Department of Surgery, The University of Iowa Carver College of Medicine, Iowa City, IA USA; 2 Free Radical and Radiation Biology Program, Department of Radiation Oncology, The University of Iowa Carver College of Medicine, Iowa City, IA USA; 3 University of Iowa Hospitals and Clinics, 1528 JCP, 200 Hawkins Drive, Iowa City, IA 52242 USA

**Keywords:** Cancer metabolism, Glycobiology

## Abstract

At pharmacological levels, ascorbate (P-AscH^-^) acts as a pro-oxidant by generating H_2_O_2_, depleting ATP in sensitive cells leading to cell death. The aim of this study was to determine the role of ATP production by oxidative phosphorylation or glycolysis in mechanisms of resistance to P-AscH^–^induced cell death. Pancreatic cancer cells were used to generate ρ^0^ cells by mitochondrial overexpression of the Y147A mutant uracil-N-glycosylase or Herpes Simplex Virus protein. The ρ^0^ phenotype was confirmed by probing for mitochondrial DNA, mitochondrial DNA-encoded cytochrome c oxidase subunit 2, and monitoring the rate of oxygen consumption. In ρ^0^ cells, glycolysis accounted for 100% of ATP production as there was no mitochondrial oxygen consumption. Even though the activities of H_2_O_2_-removing antioxidant enzymes were similar in both the parental and ρ^0^ clones, P-AscH^-^ -induced clonogenic cell death in ρ^0^ cells showed more resistance than the parental cell line. In addition, P-AscH^-^ induced more DNA damage and more consumption of NAD^+^ and greater decreases in the production of ATP in the parental cell line compared to the ρ^0^ cells. Thus, cancer cells that largely use oxidative phosphorylation to generate ATP may be more sensitive to P-AscH^-^ compared with cells that are glycolysis-dependent.

## Introduction

Encouraging results on the use of intravenous, high dose vitamin C as a therapy for cancer were first reported in the 1970s^1,2^. More recently, there has been renewed interest in the use of pharmacological ascorbate (P-AscH^-^, intravenous high dose vitamin C) to treat a variety of malignancies^3–5^. The cytotoxicity of P-AscH^-^ centers on the generation of H_2_O_2_ by ascorbate upon its oxidation. In the presence of catalytic metals (e.g. Fe^3+^/Fe^2+^), P-AscH^-^ readily oxidizes producing high fluxes of H_2_O_2_ in the extracellular space of tumors. This extracellular H_2_O_2_ can readily enter cells via peroxiporins in the plasma membrane leading to oxidative damage to tumor cells. In general, non-malignant cells are less sensitive to P-AscH^-^ and H_2_O_2_ both in vivo and in vitro^6,7^. P-AscH^-^ may deplete ATP by three different mechanisms. First, H_2_O_2_-induced DNA damage activating PARP that catabolizes NAD + , thereby depleting substrate for NADH formation and ATP synthesis. Secondly, H_2_O_2_ is catabolized by concurrent oxidation of glutathione (GSH) to glutathione disulfide (GSSG). To reduce GSSG back to GSH, glutathione disulfide reductase utilizes electrons from NADPH. Glucose that is used to reduce NADP^+^ to NADPH cannot be used for glycolysis or NADH production, thus ATP generation can be compromised. Finally, H_2_O_2_ can directly damage mitochondria leading to decreased production of ATP from oxidative phosphorylation. Cells may adapt alternative metabolic pathways, such as increasing glycolytic activity, to maintain their supply of stored energy when mitochondrial oxidative phosphorylation rates decrease, such as in hypoxia. However, increased dependence on glycolysis is associated with reduced sensitivity to anticancer treatments, *i.e.* resistance. To determine the role of mitochondrial ATP production in P-AscH^–^induced cytotoxicity, we generated mitochondrial respiratory defective clones (ρ^0^ cells) of pancreatic cancer cells. These cells also allowed us to determine if glycolysis-dependent ATP production can contribute to resistance to P -AscH^-^.

Cells depleted of mitochondrial DNA (ρ^0^ cells) are important tools for investigating mitochondrial pathways and for understanding nuclear and mitochondrial DNA interaction. Methods to generate ρ^0^ cells include chemical treatments, targeting nucleases, or manipulation of regulatory proteins^8^. The first ρ^0^ cells were generated using the intercalating dye ethidium bromide, which induced mitochondrial respiratory deficiencies in yeast cells. These mutants demonstrated less buoyant density (ρ-) of their mitochondrial DNA, hence the ρ^0^ cells^9–11^. The first animal ρ^0^ cells were developed from chicken embryo fibroblasts^11^, which required exogenous sources of pyrimidine (uridine) and pyruvate to survive and proliferate. Pyrimidine was necessary because an inner mitochondrial membrane protein dihydroorotate dehydrogenase, which requires mitochondrial electron transport for normal function, and is necessary for pyrimidine synthesis^12^. Although ρ^0^ cells generate pyruvate from glycolytic metabolism of glucose, these cells may also require an exogenous source of pyruvate. Pyruvate appears to be necessary due to its reduction to lactate through the activity of lactate dehydrogenase; this reduction utilizes the excess NADH that can accumulate in ρ^0^ cells due to the absence of a functional respiratory chain^13^.

In the present study pancreatic cancer ρ^0^ cells were generated using plasmids as described by Alexeyev et al.^14^ with the MIA PaCa-2 human pancreatic cancer cell as the host. We confirmed that these clones did not produce ATP from oxidative phosphorylation in mitochondria. We then used these ρ^0^ clones as a tool to examine mechanisms of resistance to P-AscH^-^ and the role of ATP production by oxidative phosphorylation or glycolysis have on P-AscH^–^induced cell death.

## Results

### Generation and characterization of ρ^0^ cells

Mitochondrial expression of DNA repair enzymes *E. coli* exonuclease III (ExoIII), mutant Y147A human uracil-N-glycosylase (mUNG1) or Herpes Simplex Virus 1 (HSV-1) protein UL12.5M185 leads to mtDNA depletion in human cervix carcinoma HeLa cells, NIH Swiss mouse embryo (NIH3T3) cells, and rat C6 glioma cells^14^. Using the human pancreatic carcinoma cell line MIA PaCa-2 as host cells, we transfected plasmids pMA3790 and pMA4008 that contain the gene inserts mUNG1 and HSV-1 UL12.5M185, respectively. Single cell clones were selected by sorting GFP positive cells into 96-well tissue culture plates using flow cytometry. Mitochondrial DNA-depleted ρ^0^ cells were confirmed with western blotting, PCR, and Seahorse XF96 analyzer.

**Figure 1:**
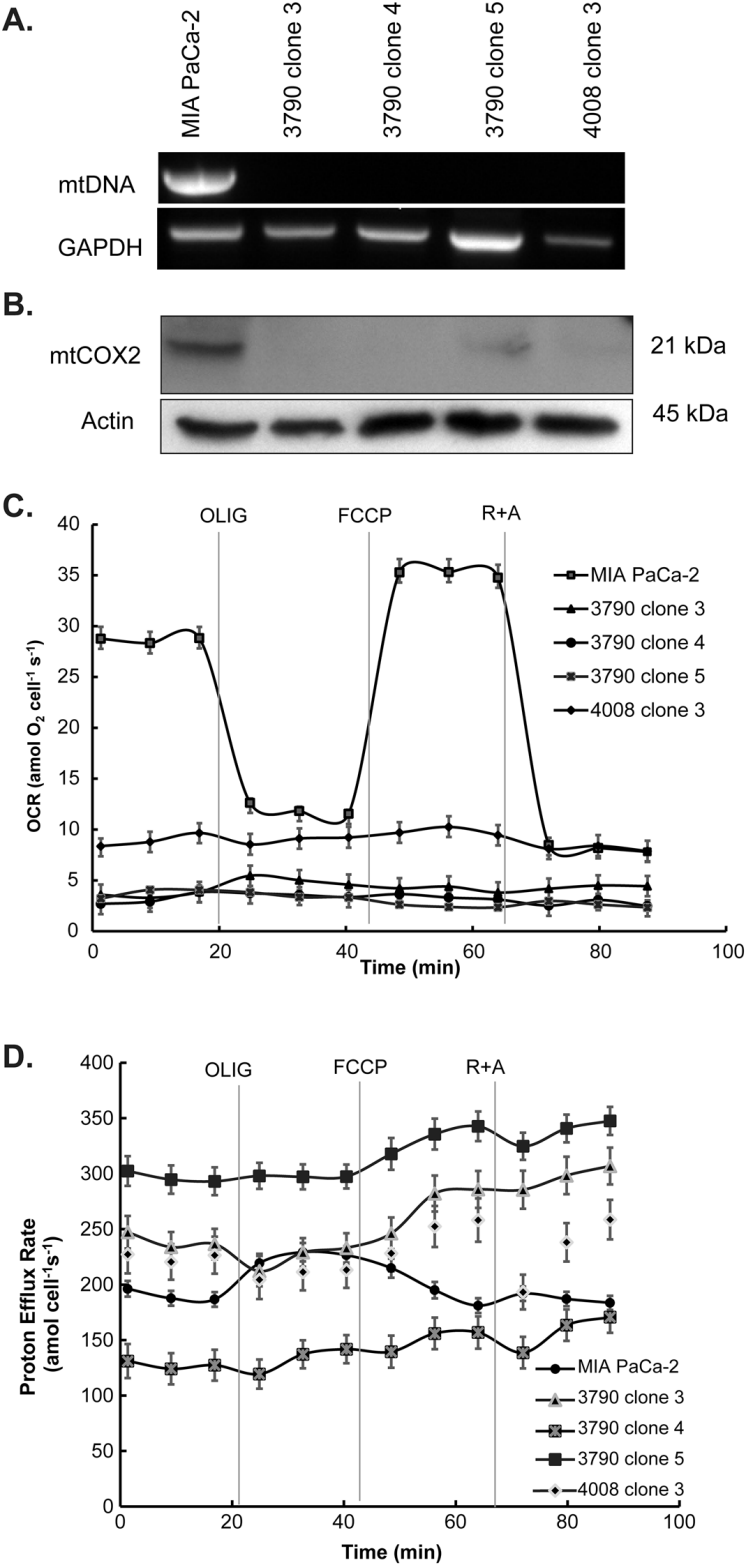
Characterization of ρ^0^ cells. (**A**) PCR analyses demonstrate intact mtDNA in the wild-type MIA PaCa-2 cell line but depletion of mtDNA in ρ^0^ cells. (**B**) Western blot analyses demonstrate expression of the mitochondrial DNA encoded protein cytochrome c oxidase subunit 2 (mtCOX2) is absent in the ρ^0^ clones. (**C**) Oxygen consumption rate (OCR) measured in MIA PaCa-2 and ρ^0^ clones under basal conditions and following the injection of oligomycin A (OLIG), carbonyl cyanide-p-trifluoromethoxyphenyl-hydrazon (FCCP), and rotenone + antimycin A (R + A). Note the lack of changes in OCR with the various metabolic inhibitors in ρ^0^ cells. (**D**) Proton Efflux Rates (PER) of MIA PaCa-2 and ρ^0^ cells. The parental cell line as well as ρ^0^ clones have differing glycolytic rates as reflected by proton efflux rates. Note that the parental cell line shows increased PER after oligomycin treatment to compensate for lost ATP production from oxidative phosphorylation. The ρ^0^ clones have no response upon addition of oligomycin, consistent with the lack of oxidative phosphorylation.

First, to verify the mtDNA depletion, total cellular DNA was extracted and PCR was performed using a human mtDNA specific primer^15,16^; the expression of nuclear DNA encoded GAPDH was used as a control. The agarose gel electrophoresis of PCR products demonstrated depletion of mtDNA in ρ^0^ clones 3790 clone 3, 3790 clone 4, 3790 clone 5, and 4008 clone 3 when compared to the parental cell line MIA PaCa-2 (Fig. 1A). Further characterization with immunoblots for cytochrome c oxidase subunit 2 (COX2), which is encoded by mitochondrial DNA, demonstrated that this protein is present in parental MIA PaCa-2, but not in ρ^0^ cells (Fig. 1B).

Mitochondrial DNA encodes 13 protein subunits of the mitochondrial respiratory chain; thus, depletion of mtDNA will result in respiratory deficiency. If cells do not have functional mitochondria for the production of ATP, then there should be no mitochondrial-associated oxygen consumption. We measured the rate of cellular oxygen consumption (OCR) and proton efflux rate (PER) in live cells using the Seahorse mitochondrial stress test assay to screen and confirm the generation of true ρ^0^ cells, Fig. 1C. As seen in Fig. 1C, MIA PaCa-2 cells had a baseline OCR of 28.8 ± 1.1 amol cell^−1^ s^−1^, which was significantly decreased after inhibition of ATP synthase (complex V) by treatment with oligomycin A. Addition of the uncoupler FCCP induced maximal OCR, while treatment with rotenone and antimycin A (inhibitors of complex I and III) decreased OCR to a low rate that represents non-mitochondrial oxygen consumption. The ρ^0^ clones have a lower basal OCR (less than 10 amol cell^-1^ s^-1^) than MIA PaCa-2; importantly, they did not respond to the respiratory modulators oligomycin, FCCP, or rotenone/antimycin A (Fig. 1C). Thus, as expected for true ρ^0^ cells, there is no mitochondrial oxygen consumption contributing the observed basal oxygen consumption.

The proton efflux rate (PER) contains information on both respiration and glycolysis. MIA PaCa-2 cells had a baseline PER of 196 ± 1 amol cell^−1^ s^−1^, Fig. 1D. As expected, treatment with oligomycin A inhibited oxidative phosphorylation in mitochondria of MIA PaCa-2 cells with a compensatory increase in glycolysis, leading to an increase in PER to 229 ± 33 amol cell^−1^ s^−1^. The addition of FCCP, rotenone, and antimycin A resulted in a decrease in PER to below baseline levels; PER estimates following addition of FCCP cannot be used quantitatively as FCCP complicates PER calculations due to its proton ionophoric nature as well as the enhanced contributions of CO_2_ to PER when inducing maximal respiration. All the ρ^0^ clones used lacked responses to the mitochondrial inhibitors but had variable baseline PERs with the 3790 clone 5 having the highest PER at 302 ± 1 amol cell^−1^ s^−1^ and 3790 clone 4 having the lowest PER at 131 ± 1 amol cell^−1^ s^−1^. These data are compatible with a lack of mtDNA and lack of functional respiratory chains in the ρ^0^ clones, and increased reliance on glycolysis to generate ATP. However, 3790 clone 6 demonstrated the presence of mitochondrial DNA (Supporting Information Fig. S1A) and residual mitochondrial function (Supporting Information Fig. S1B) and thus was excluded from detailed studies as they are not true ρ^0^ cells. For experiments described below, we only used clones that demonstrated the ρ^0^ phenotype as confirmed by Western blotting, PCR, and Seahorse XFe96 analyzer.

### Characterization of ρ^0^ cells with glycolytic rate and ATP rate assays

**Figure 2:**
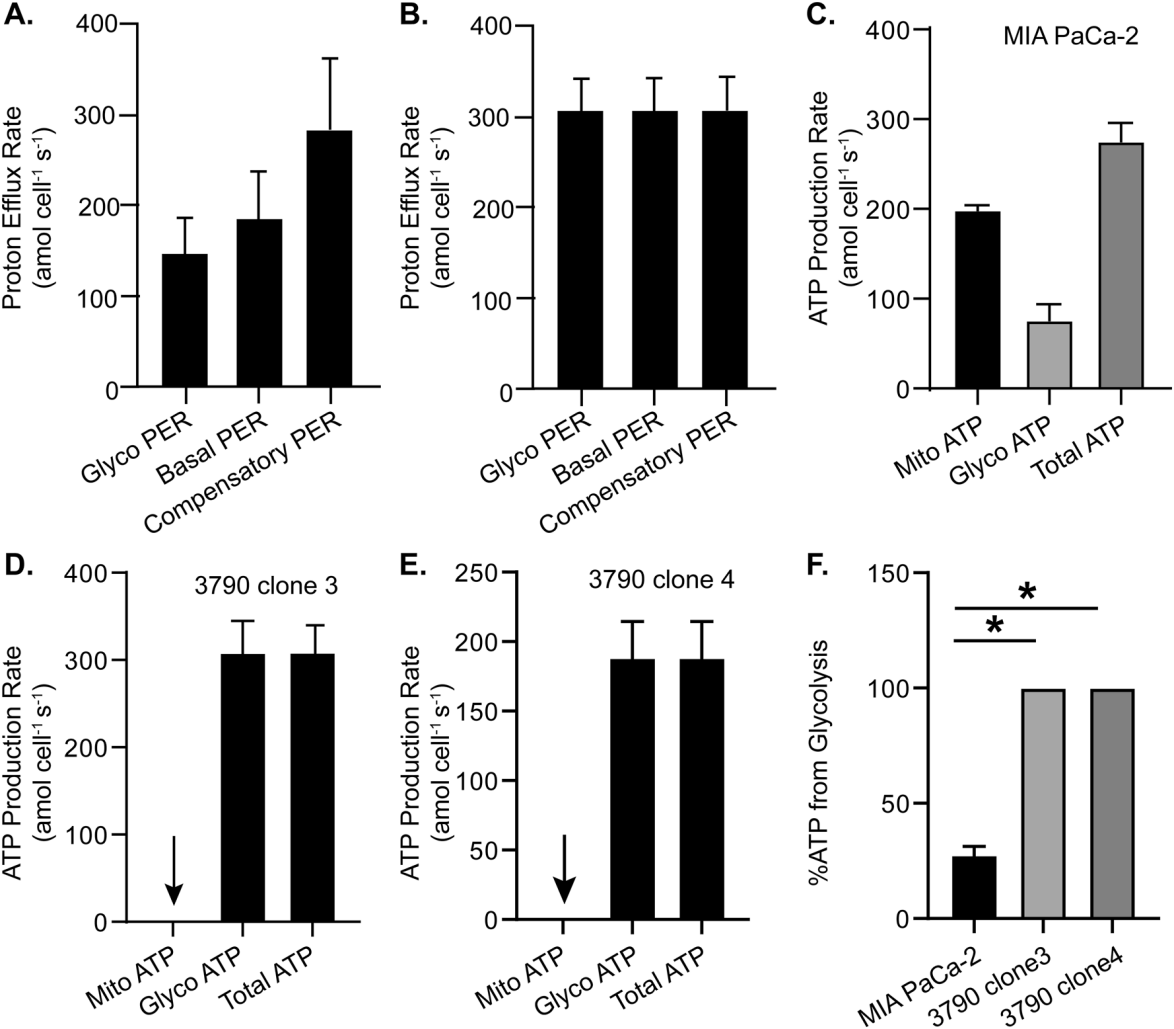
Glycolytic contribution to PER in MIA PaCa-2 and ρ^0^ cells. MIA PaCa-2 and ρ^0^ cells were seeded into a Seahorse 96-well XF Cell Culture Microplate. After 48 h, extracellular acidification rate (ECAR) and oxygen consumption rate (OCR) were measured. GlycoPER, basal PER and compensatory PER were calculated and normalized to cell number and expressed as amol cell^−1^ s^−1^ ^36^. (**A**) GlycoPER, basal PER, and compensatory PER of MIA PaCa-2 cells (*n* = 3, mean ± SEM). (**B**) GlycoPER, basal PER and compensatory PER of 3790 clone 3 (*n* = 3, mean ± SEM). (**C**) Contributions to the total rate of production of ATP from mitochondrial and glycolytic sources in MIA PaCa-2 cells (*n* = 3, mean ± SEM). (**D**) The rates of production of ATP in the 3790 clone 3 demonstrates that 100% of ATP is derived from glycolytic sources (*n* = 5, mean ± SEM) (arrow, no mitochondrial ATP production). (**E**) The rate of production of ATP in the 3790 clone 4 demonstrates that 100% of ATP is derived from glycolysis (*n* = 5, mean ± SEM) (arrow, no mitochondrial ATP production). (**F**) % ATP from glycolysis demonstrates that in the MIA PaCa-2 cells 27 ± 4% ATP is derived from glycolytic sources while both ρ^0^ clones rely 100% on glycolysis (*n* = 3, mean ± SEM; *p < 0.05, unpaired *t* test).

In addition to the mitochondrial stress test, we employed the glycolytic rate assay, which provides a more rigorous estimate of glycolysis in live cells. MIA PaCa-2 parental cells had a basal PER of 185 ± 53 amol cell^−1^ s^−1^; however, when the CO_2_ contribution from respiration is accounted for in PER, the glycolytic rate assay yielded a corrected estimate of the glycolytic rate, *i.e.* glycoPER, of 145 ± 43 amol cell^−1^ s^−1^. Thus, glycolysis is contributing ≈78% of basal PER and mitochondrial derived CO_2_ is contributing ≈22% (Fig. 2A). In contrast, 100% of basal PER was from glycolysis in the 3790 clone 3 (Fig. 2B) and all other ρ^0^ clones (Supporting Information Fig. S2A), indicating there were no functional mitochondrial electron transport chains and no mitochondrial derived acidification due to CO_2_. Thus, in ρ^0^ clones PER = glycoPER.

The information above can be used to quantify the rate of ATP production from glycolysis and mitochondria. The ATP rate assay demonstrated that in MIA PaCa-2 cells the rate of production of ATP from glycolysis was 76 ± 18 amol cell^−1^ s^−1^, the mitochondrial ATP production rate was 199 ± 5 amol cell^−1^ s^−1^, yielding a total rate of production of ATP of 276 ± 20 amol cell^−1^ s^−1^ (Fig. 2C). Thus, 27 ± 4% of the production of ATP was from glycolysis with the rest being generated from mitochondrial oxidative phosphorylation. In the four ρ^0^ cell lines, total ATP production rates ranged from to 308 ± 36 amol cell^−1^ s^−1^ (3790 clone 3) (Fig. 2D) to 161 ± 5 amol cell^−1^ s^−1^ (4008 clone 3) (Fig. 2E and Supporting Information Fig. S2B) with 100% of ATP production coming from glycolysis (Fig. 2F and Supporting Information Fig. S2B). These results again indicate that ρ^0^ cells do not have functional electron transport in their mitochondria to support production of ATP by oxidative phosphorylation.

In addition to the ATP rate assay, we also measured steady-state levels of intracellular ATP in MIA PaCa-2 and two ρ^0^ clones (3790 clone 3 and 3790 clone 4). The intracellular levels of the 3790 clone 3 (3.8 ± 1.0 mM) and the 3790 clone 4 (2.2 ± 1.0 mM) were significantly less (*p < 0.05) than parental MIA PaCa-2 cells (6.2 ± 1.0 mM). The lower levels of intracellular ATP might be due to increased energy demand in ρ^0^ cells.

### Ultra-structural studies with transmission electron microscopy

Abnormalities of mitochondrial ultrastructure have been described previously in ρ^0^ cells from different tissue origins^17,18^. To study the morphological changes seen in images of pancreatic cancer cells from transmission electron microscopic, we compared two ρ^0^ clones with the parental MIA PaCa-2 cells. As shown in Supporting Information Fig. S3, mitochondria of MIA PaCa-2 had relatively regular cristae, ρ^0^ 3790 clone 3 had enlarged mitochondria with complete loss of cristae, while 3790 clone 4 had smaller mitochondria with abnormally arranged cristae or complete loss of cristae. The abnormality in mitochondrial ultrastructure is similar to those observed previously^17,18^.

### Effects of pharmacological ascorbate (P-AscH^-^)

**Figure 3:**
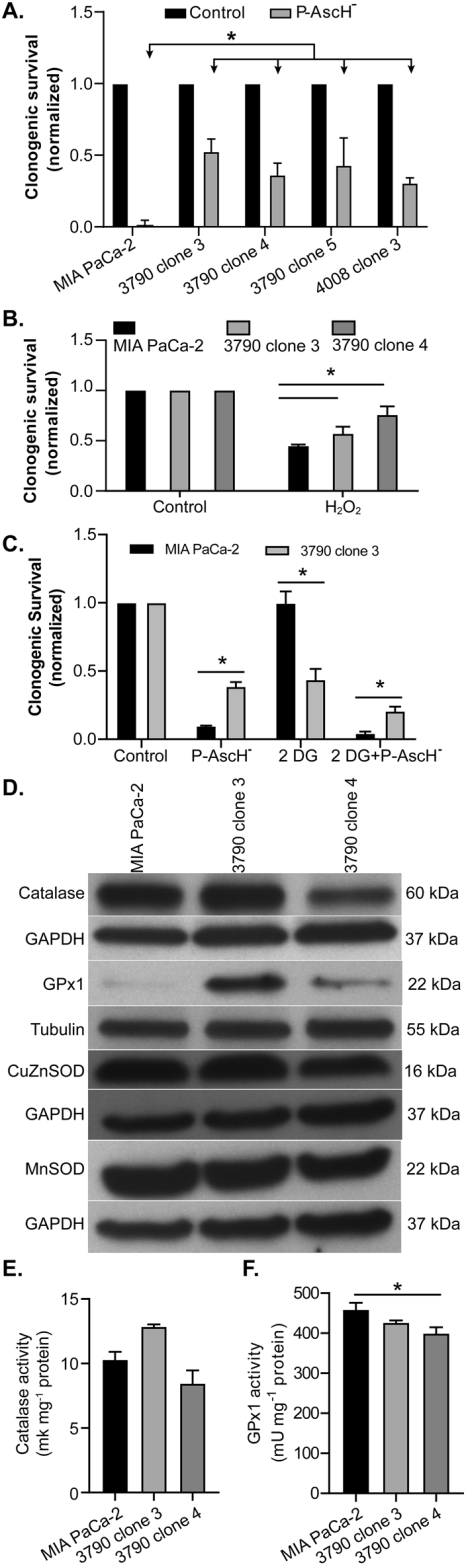
ρ^0^ cells lacking mitochondrial oxidative phosphorylation are resistant to P-AscH^-^. (**A**) Following treatment with P-AscH^-^, (9 pmol cell^−1^ for 1 h), clonogenic cell survival of MIA PaCa-2 and ρ^0^ cells were evaluated. Results demonstrate resistance as seen by clonogenic assays in all ρ^0^ clones (*n* = 3, mean ± SEM; *p < 0.05, one-way ANOVA Tukey’s multiple comparison test). Alterations in protein expression and activity of antioxidant enzymes in MIA PaCa-2 and ρ^0^ cells. (**B**) Following treatment with 20 µM H_2_O_2_, clonogenic survival of MIA PaCa-2 and ρ^0^ cells demonstrate resistance in the ρ0 clones (*n* = 3, mean ± SEM; *p < 0.05, one-way ANOVA Tukey’s multiple comparison test). (**C**) MIA PaCa-2 and the p^0^ 3790 clone 3 were treated with 2-deoxyglucose (20 mM) with and without P-AscH^-^ (2 mM) (n = 3, mean ± SEM; *p < 0.05, one-way ANOVA Tukey’s multiple comparison test). (**D**) Western blot analyses show the levels of catalase, GPx1, CuZnSOD (SOD1) and MnSOD (SOD2) in the MIA PaCa-2 and two ρ^0^ clones. The blots depicted are representative of three separate experiments. There were minimal changes in the expression of SOD protein in the parental or ρ^0^ clones. (**E**) Catalase activity of MIA PaCa-2 and ρ^0^ cells (*n* = 3, mean ± SEM; *p* > 0.05, MIA PaCa-2 *vs*. 3790 clone 3 or vs. 3790 clone 4; one-way ANOVA Tukey’s multiple comparison test). (**F**) GPx1 activity was decreased in 3790 clone 4 compared to MIA PaCa-2 (*p < 0.05, Mean ± SEM, *n* = 3; one-way ANOVA Tukey’s multiple comparison test).

Depletion of mitochondrial DNA in ρ^0^ cells has been found to enhance resistance to cell death induced by ionizing radiation, H_2_O_2_, and other prooxidants^16,19^. We exposed MIA PaCa-2 and the ρ^0^ clones to P-AscH^-^ (2 mM, 9 pmol cell^−1^ for 1 h) in DMEM-10% FBS without pyruvate (pyruvate can scavenge H_2_O_2_^20,21^) and determined clonogenic survival. As seen in Fig. 3A, P-AscH^-^ significantly decreased colony formation in MIA PaCa-2 cells compared to the four ρ^0^ clones (*p < 0.05). Because P-AscH^-^ induces cell death via formation of extracellular H_2_O_2_, we then exposed the MIA PaCa-2 parental cell line and the 3790 clone 3 and 3790 clone 4 to H_2_O_2_ (20 µM). Data in Fig. 3B demonstrate that this dose of peroxide decreased clonogenic survival in the parental cell line to a significantly greater degree than with either of the two ρ^0^ clones (*p < 0.05). A more direct measurement of H_2_O_2_ levels was performed using the using both the oxidation sensitive and insensitive DCF-DA fluorescent dyes with flow cytometric analysis; we observed no differences in DCF-DA fluorescence (presumably due to H_2_O_2_) after 1 h treatment of P-AscH^-^ (10 pmol/cell) in the parental cell lines *vs.* the ρ^0^ clones (Supporting Information Fig. S4).

We hypothesized that if 100% of the production of ATP was from glycolysis in the ρ^0^ clones, as seen in Fig. 2F, then they would be more sensitive to the glucose analog 2-deoxyglucose 2DG), which acts as a chemical inhibitor of glucose metabolism. Consistent with data in Fig. 3A, the 3790 clone 3 was still resistant to P-AscH^-^ compared to the MIA PaCa-2 parental cell line (*p < 0.05) (Fig. 3C). In addition, the ρ^0^ clone was more sensitive to 2DG compared to the MIA PaCa-2 cell line *p < 0.05) (Fig. 3C). The combination of P-AscH^-^ with 2DG further decreased clonogenic survival with the ρ^0^ clone being more resistant.

Changes in catalase activity could contribute to the resistance of P-AscH^-^ in the ρ^0^ clones. Catalase is the main antioxidant enzyme that detoxifies H_2_O_2_ generated by P-AscH^-^. In fact, catalase levels may be a possible indicator for how tumors will respond to P-AscH^-^^22,23^. A previous study by Vergani et al. demonstrated that loss of functional mitochondria, the major cellular site for reactive oxygen species production, may cause alteration in enzymatic and non-enzymatic antioxidant defenses in ρ^0^ cells derived from bone, muscle, and lung^24^. Here we show that the loss of mtDNA results in minimal changes in catalase in 3790 clone 3 and 3790 clone 4 compared to the parental cells (Fig. 3D). GPx1 expression was increased in the clones compared to the parental cell lines (Fig. 3D). Although located in the mitochondrial matrix, MnSOD (SOD2) protein is encoded by the SOD2 gene in the nucleus; the deletion of mtDNA had minimal effects on its expression. In addition, nuclear encoded CuZnSOD (SOD1) expression did not appear to be affected in the two ρ^0^ clones (Fig. 3D). The expression of mRNA for antioxidant enzymes does not necessarily reflect antioxidant enzyme protein or activity^25^. Even increased immunoreactive antioxidant enzyme protein is not necessarily indicative of increased activity. Thus, activity is believed to be the most important parameter determining the biological impact of antioxidant enzymes. Catalase activities in the two ρ^0^ cell clones were similar to the parental cell line (Fig. 3E). There was a decrease of GPx1 activity in 3790 clone 4 cells compared to the MIA PaCa-2 parental cell line (Fig. 3F, * *p* < 0.05). Previous studies from our laboratory have demonstrated that an increase in catalase activity of > 150-fold from typical levels is needed to reverse P-AscH^-^ induced toxicity ^26^. Thus, the minimal decrease is GPx activity in this clone would not significantly affect the resistance to P-AscH^-^. Thus, the resistance of ρ^0^ clones to P-AscH^–^induced H_2_O_2_ formation, compared to MIA PaCa-2 cells, is unlikely due to differences in catalase or GPx-1 activities.

**Figure 4:**
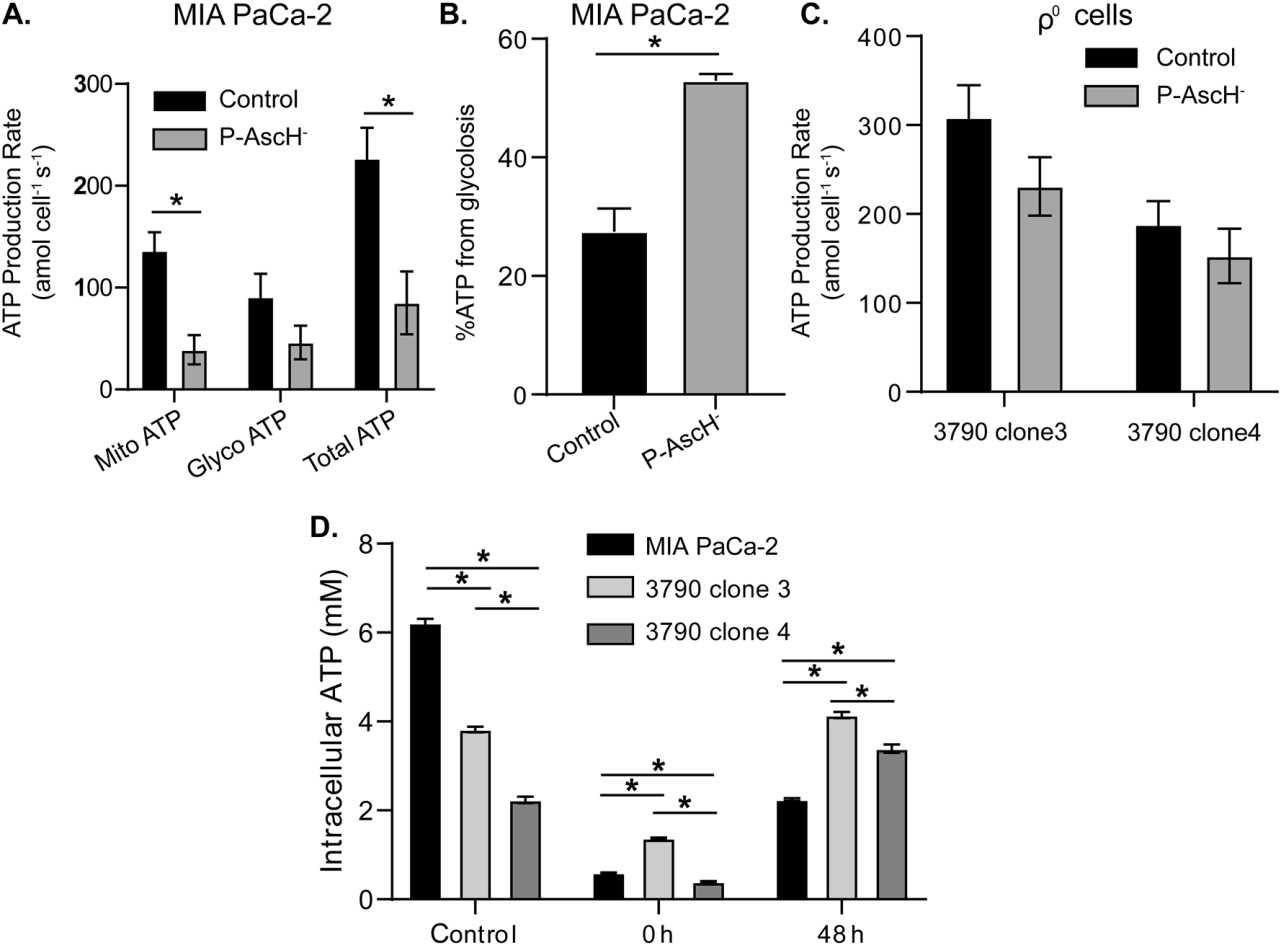
Changes in ATP in MIA PaCa-2 and ρ^0^ cells following treatment with P-AscH^-^. MIA PaCa-2 and ρ^0^ clones were exposed to P-AscH^-^ (7 pmol cell^−1^ for 1 h) and the rates of production of glycolytic ATP, mitochondrial ATP, and total ATP were measured 48 h later. Rates are normalized to cell number and expressed as amol cell^−1^ s^−1^^41^. (**A**) Rates of production ATP from mitochondria and total ATP of MIA PaCa-2 cells were decreased 48 h after exposure to P-AscH^-^ while glycolytic ATP production was less effected (*n* = 3, mean ± SEM; *p < 0.05, unpaired t-test). (**B**) The percent contribution of glycolysis to the production of ATP in MIA PACa-2 cells increased after exposure to P-AscH^-^ (*n* = 3, mean ± SEM; *p < 0.05, unpaired *t* test). (**C**) Compared to the significant decrease in the rate of production of ATP seen in panel A, the rate of production of ATP in ρ^0^ cells (3790 clone 3, 3790 clone 4) did not change significantly (*n* = 3, mean ± SEM). (**D**) Steady-state levels of intracellular ATP of MIA PaCa-2 and ρ^0^ cells decreased significantly after P-AscH^-^. There was a significant decrease in ATP levels in MIA PaCa-2 cells compared to the ρ^0^ cells at 1 h after exposure P-AscH^-^ (*n* = 3, mean ± SEM, *p < 0.05, one-way ANOVA Tukey’s multiple comparison test). At 48 h, intracellular ATP levels are significantly increased in ρ^0^ cells compared to MIA PaCa-2 cells (*n* = 3, mean ± SEM, *p < 0.05, one-way ANOVA Tukey’s multiple comparison test).

Previously we demonstrated that H_2_O_2_ generated by the oxidation of P-AscH^-^ causes DNA damage, leading to activation of PARP1, consumption of NAD^+^, and subsequent temporary depletion of intracellular ATP^6^. Using the Seahorse ATP rate assay 48 h after exposure to P-AscH^-^ (7 pmol cell^−1^) (Fig. 4), the rate of mitochondrial ATP production in MIA PaCa-2 cells was decreased significantly (*p* < 0.05) from 136 ± 18 to 39 ± 14 amol cell^-1^ s^-1^; glycolytic ATP production decreased from 76 ± 18 to 46 ± 17 amol cell^−1^ s^−1^ in MIA PaCa-2 cells (Fig. 4A). Although the overall rate of production of ATP decreased after treatment with P-AscH^-^, it appeared that 53 ± 1% of ATP production was from glycolysis compared to 27 ± 4% in MIA PaCa-2 control cells (Fig. 4B). As shown in Fig. 4C, 48 h after treatment with P-AscH^-^ the rate of glycolytic production of ATP decreased from 308 ± 36 to 231 ± 33 amol cell^−1^ s^−1^ in the 3790 clone 3 cells (*p* = 0.14) and from 188 ± 26 to 153 ± 31 amol cell^-1^ s^-1^ in the 3790 clone 4 cells (*p* = 0.4). In general, the ρ^0^ cells maintain a higher rate of total ATP production following treatment with P-AscH^-^ than MIA PaCa-2 and do so exclusively through glycolysis. MIA PaCa-2 cells seem to have a greater reliance on glycolysis to produce ATP 48 h after exposure to P-AscH^-^.

Previously we demonstrated that treatment with P-AscH^-^ for 1 h causes immediate depletion of intracellular ATP in MIA PaCa-2 cells^6^. Here we have found that treatment with P-AscH^-^ causes a significant decrease in steady-state levels of ATP in both the parental cell line and the ρ^0^ clones (Fig. 4D). However, 48 h after exposure to P-AscH^-^, the ATP levels in the ρ^0^ clones were significantly greater than the MIA PaCa-2 cells, which may confer partial resistance to P-AscH^–^induced decreases in clonogenic survival (Fig. 4D).

### Alterations in P-AscH^-^ induced DNA damage and consumption of NAD^+^ in ρ^0^ cells

**Figure 5:**
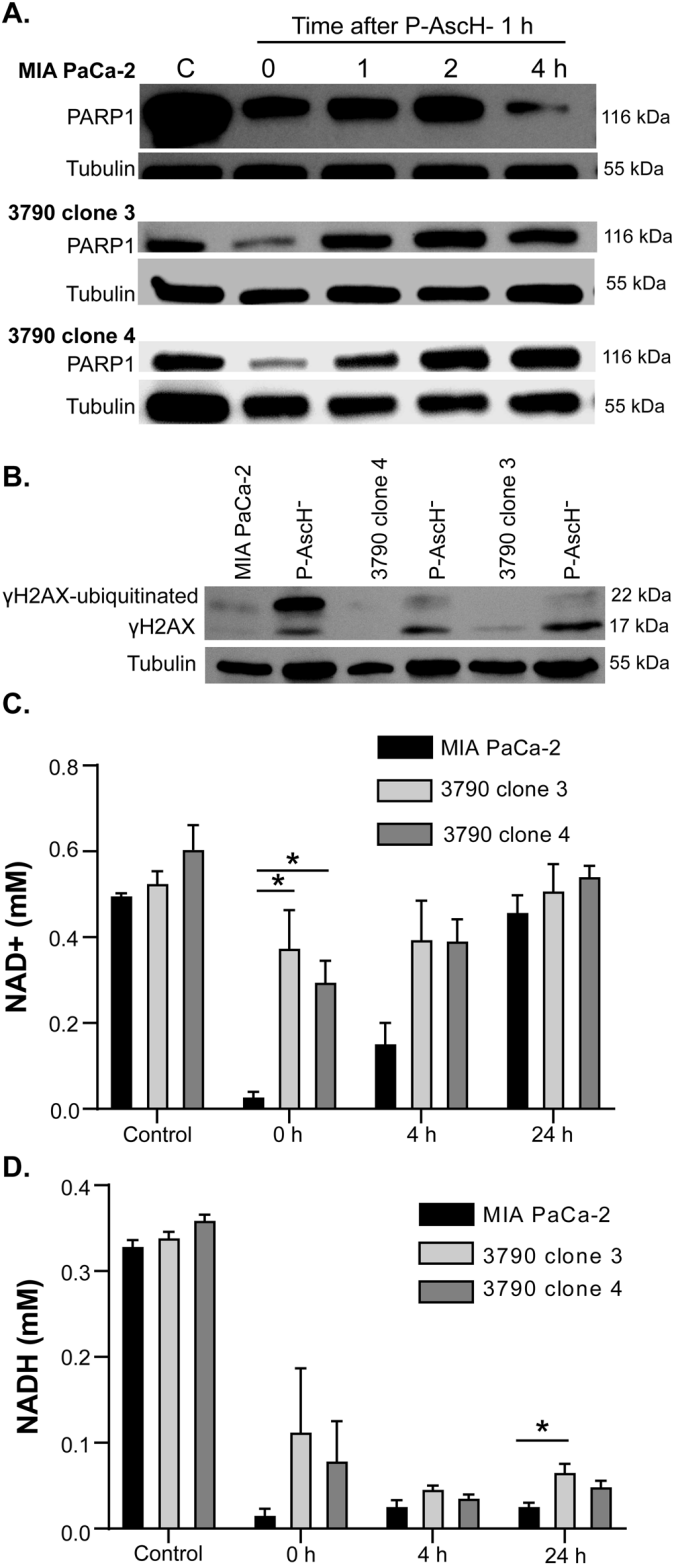
P-AscH^-^ induced DNA damage and NAD^+^/NADH consumption is reduced in ρ^0^ cells. (**A**) Western blot analyses demonstrate decreases in PARP1 at 4 h following exposure to P-AscH^-^ (10 pmol cell^−1^ for 1 h) in MIA PaCa-2 cells and minimal changes in the two ρ^0^ clones. (**B**) Western blot analyses demonstrate increases in phosphorylated γH2AX in MIA PaCa-2 cells following P-AscH^-^ (14 pmol cell^−1^) treatment at for 1 h. In contrast the two ρ^0^ clones had less phosphorylated γH2AX when compared to the parental cell line while all 3 clones had similar levels of γH2AX following exposure to P-AscH^−^. (**C**) Steady-state levels of NAD^+^ are similar in the MIA PaCa-2 and ρ^0^ clones. However, there is greater loss of NAD^+^ immediately after P-AscH^-^ treatment (0 h) in the MIA PaCa-2 cells (exposure: 6 pmol cell^−1^ for 1 h) compared to the ρ^0^ clones. Levels of NAD^+^ nearly recover to pretreatment steady-state levels 24 h after exposure to P-AscH^-^ (*n* = 3, means ± SEM; *p < 0.05, one-way ANOVA Tukey’s multiple comparison test). (**D**) Steady-state levels of NADH are similar in the MIA PaCa-2 and ρ^0^ clones. However, there is greater loss of NADH immediately after treatment with P-AscH^-^ (6 pmol cell^−1^ for 1 h) in MIA PaCa-2 cells than in ρ^0^ cells. Steady-state levels of NADH do not recover to pretreatment levels (*n* = 3, mean ± SEM; *p < 0.05, one-way ANOVA Tukey’s multiple comparison test).

The H_2_O_2_ generated by P-AscH^-^ causes single and double strand breaks with PARP1 involved in the repair of both pathways^27^. PARP1 activation consumes large amounts of NAD^+^^28^. As shown in Fig. 5A, P-AscH^-^ (10 pmol cell^−1^) treatment for 1 h induced initial decreases of PARP1 in both MIA PaCa-2 and two ρ^0^ clones. However, in MIA PaCa-2 cells, PARP1 expression decreased at 4 h, compared to the two ρ^0^ cells where there was sustained PARP1 expression, indicating a differential response following DNA damage. γH2AX, a marker of DNA double strand breaks^29^, was increased in MIA PaCa-2 cells 1 h after treatment with P-AscH^-^ (14 pmol cell^-1^) (Fig. 5B). This P-AscH^–^induced increase in γH2AX was also observed in the 3790 clone 4 and the 3790 clone 3 but not to the same degree. Previous studies have demonstrated that ubiquitinated γ-H2AX is the predominant form accounting up to 80–90% of total γ-H2AX in response to mechanistically distinct DNA double strand break inducers^41^. Figure 5C shows significant differences in the steady-state levels of NAD^+^ in the ρ^0^ clones following treatment with P-AscH^-^. In the MIA PaCa-2 parental cell line, 90% of NAD^+^ appears to be consumed by 1 h, while in the 3790 clone 3 only 36% of NAD^+^ is consumed and in the 3790 clone 4 only 51% of NAD^+^ is consumed at 1 h indicating the ρ^0^ cells are more resistant to changes in the steady-state levels of NAD^+^ due to exposure to P-AscH^-^. Finally, more NADH is consumed or its steady-state level decreases in MIA PaCa-2 than in the two ρ^0^ clones following treatment with P-AscH^-^ (Fig. 5D). Thus, upon exposure to P-AscH^-^ ρ^0^ cells appear to have less DNA damage and associated downstream responses.

## Discussion

Compared to MIA PaCa-2 cells, ρ^0^ cells were more resistant to P-AscH^–^induced cytotoxicity due to H_2_O_2_. There was less DNA damage leading to a smaller loss of NAD^+^ in ρ^0^ cells following exposure to P-AscH^-^. In addition, P-AscH^-^ significantly decreased the rate of production of ATP in MIA PaCa-2 cells compared to the ρ^0^ clones 48 h after exposure, indicating a decreased demand or limited capacity to generate ATP in MIA PaCa-2 compared to the two ρ^0^ cell lines. The production of ATP seems to shift toward glycolysis in MIA PaCa-2 cells 48 h after exposure to P-AscH^-^.

Our present study demonstrates the successful generation of ρ^0^ cells by mitochondrial overexpression of the Y147A mutant uracil-N-glycosylase (mUNG1) or Herpes Simplex Virus protein UL12.5M185. Absence of mitochondrial DNA was confirmed with: PCR probing for mtDNA; the loss of expression of mtDNA-encoded COX2; and loss of mitochondrial oxygen consumption as well as the lack of responses to mitochondrial inhibitors. In these same ρ^0^ cells 100% of the rates of basal proton efflux and 100% of the production of ATP are from glycolysis. The ρ^0^ clones are: more resistant to clonogenic cell death; NAD^+^ consumption (as seen by smaller changes in steady-state levels)’ and less DNA damage compared to the parental cell line after exposure to P-AscH^-^.

The first human cells lacking mtDNA were generated by long-term exposure to low concentrations of ethidium bromide^30^. However, depletion of mitochondrial DNA using EtBr has been shown to induce multidrug resistant genes and has other mutagenic effects in cells, including nuclear targets^17,31^. To overcome these disadvantages, mitochondrially targeted DNA repair enzymes have been modified to generate ρ^0^ cells^14,17^ allowing mtDNA selectivity. Kukat et al*.* generated human osteosarcoma cells 143B.TK^-^ rho cells using mitochondrially targeted EcoRI restriction endonuclease^17^; the Alexeyev group employed ExoIII, mUNG1and HSV-1 UL12.5M185 protein to generate ρ^0^ cells from mouse, rat, and human cells^14,32^. These newer approaches have greater specificity and fewer off-target effects.

Previous studies from our group have demonstrated that sensitivity of tumor cells to P-AscH^−^ may be due to their lower capacity to metabolize H_2_O_2_^22^. Rate constants for removal of extracellular H_2_O_2_ (*k*_cell_) and catalase activities were determined for 15 tumor and 10 normal cell lines of various tissue types^22^. A differential in the capacity of cells to remove H_2_O_2_ was revealed, with the average *k*_cell_ for normal cells being twice that of tumor cells. The ED_50_ (50% clonogenic survival) upon exposure to P-AscH^−^ correlated directly with *k*_cell_ as well as catalase activity. In our current study, there were no significant changes in antioxidant enzymes, including catalase, that could contribute to the resistance of P-AscH^-^ in the ρ^0^ clones. These observations point to the production of ATP as a key factor in resistance of P-AscH^-^.

In the present study, MIA PaCa-2 cells were transfected with plasmids containing mUNG1 (pMA3790) or HSV-1 UL12.5M185 (pMA4008) gene to generate ρ^0^ cells. Three ρ^0^ cell lines were derived from pMA3790 and one ρ^0^ cell line from pMA4008, which confirmed that pMA3790 is very efficient at inducing the ρ^0^ phenotype^14^. These ρ^0^ cells are stable cell lines without mtDNA and mtDNA encoded proteins, the cell lines can be frozen in liquid nitrogen with growth media containing 10% DMSO. Furthermore, these four ρ^0^ cells demonstrate low OCR; this low rate of oxygen consumption was from non-mitochondrial origins, with no oxygen consumption from oxidative phosphorylation to generate ATP. With the loss a functional electron transport chain and mitochondrial ATP production, the ρ^0^ clones were found to rely on glycolysis for ATP production. The ρ^0^ clones we selected for further study each have different glycolytic and ATP production rates unique to each clone, but they all share a common trait in that their production of ATP, they are 100% dependent on glycolysis. Most importantly, the p^0^ clones were resistant to P-AscH^-^ suggesting that cancer cells that largely use oxidative phosphorylation to generate ATP may be more sensitive to P-AscH^-^ compared with cells that are glycolysis-dependent.

## Experimental procedures

### Cell culture

Human pancreatic cancer cell line MIA PaCa-2 (ATCC, CRL1420) was cultured in Dulbecco’s Modified Eagle Medium (DMEM) containing 10% fetal bovine serum (FBS). ρ^0^ cells were grown in DMEM containing 10% FBS, 50 µg/mL uridine and 1 mM sodium pyruvate. All cells were incubated in a humidified atmosphere containing 5% CO_2_ at 37 °C.

Plasmids pMA3790 and pMA4008 were created in the Mikhail Alexeyev lab^14^. pMA3790 contains gene inserts mutant Y147A human uracil-N-glycosylase (mUNG1) and Enhanced Green Fluorescent Protein (EGFP) as well as human OTC (ornithine transcarbamylase) mitochondrial targeting sequence and selectable marker Neomycin (G418). pMA4008 contains gene inserts Herpes Simplex Virus 1 (HSV-1) UL12.5M185 and EGFP. The HSV-1*UL12* gene encodes an alkaline DNase located in the nucleus; however, UL12.5M185 is a truncated version of the DNase with a methionine residue M185 that localizes to mitochondria^33^.

### Plasmid transfection

Plasmids pMA3790 and pMA4008 were obtained from Addgene (#70,110 and #70,109, respectively). Plasmids in transformed bacteria stab culture were processed by ViraQuest (Coralville, IA); purified plasmid DNA were sequenced and confirmed thereafter by the same company. Transfection of plasmids was carried out using a Lipofectamine 2000 Reagent (Life Technologies) according to the manufacturer’s directions. 72 h after transfection, EGFP positive cells were sorted into 96-well tissue culture plates containing 200 µL/well ρ^0^ growth medium with a Becton Dickinson Aria II cell sorter. Single cell clones were transferred to 60-mm dishes for further analysis 2–3 weeks later.

### Confirmation of ρ^0^ cells by PCR

Total cellular DNA was extracted with a DNeasy® Blood & Tissue Kit (QIAGEN). Amplification of mtDNA was performed using human mtDNA specific primer: Mts1 (forward) (5’-cctagggataacagcgcaat) and Mtas1 (reverse) (5’-tagaagagcgatggtgagag), which gave a 630-bp product^15,16^. Each reaction contained 50 µL of 10 × Coralload PCR buffer (*Taq* PCR Core Kit, QIAGEN), 15 ng of template DNA, 5 µM of each primer and RNase-free water. PCR parameters underwent initial denaturation for 3 min at 94 °C, then 25 cycles of denaturation for 1 min at 94 °C, annealing for 1 min at 55 °C and extension for 1 min at 72 °C, and then final extension for 10 min at 72 °C. For control, we amplified GAPDH primer (Integrated DNA Technologies): GAPDH (forward) (5’-accacagtccatgccatcac) and GAPDH (reverse) (5’-tccaccaccctgttgctgta).

### Western blotting

Cells were lysed in RIPA buffer and cell lysate pelleted by centrifugation. Protein concentrations were determined using a Bio-Rad DC Bradford Protein Assay (Bio-Rad Laboratories). Cell lysates (20 to 40 μg of protein) were electrophoresed in a Bio-Rad 4–20% Precast Gel. The proteins were electrotransferred onto a PVDF membrane, and blocked with 5% nonfat milk in 0.1% Tween-PBS (TPBS) for 60 min. The membranes were incubated with primary antibodies at 4 ˚C overnight. Horseradish peroxidase-conjugated goat anti-rabbit or goat anti-mouse was used as secondary antibody. Membranes were stained with Super Signal West Pico PLUS chemiluminescent substrate (Thermo Scientific) and exposed to Classic Blue Autoradiography Film (Molecular Technologies).

### Exposure of cells to P-AscH

When cells were at the desired log phase growth state, they were then rinsed with PBS and placed in fresh medium without pyruvate, as pyruvate reacts with H_2_O_2_. Cells were treated with ascorbate, typically some pmol/cell, from a stock solution of 1.0 mol L^−1^ ascorbate (pH 7.0), which was made under N_2_ or Argon and stored in a volumetric flask with a tight-fitting stopper at 4 °C. The concentration of ascorbate in the stock solution was verified using its UV/Vis absorbance ε_265_ = 14,500 M^-1^ cm^-1^ at 265 nm after diluting appropriately into a pH ≈6.5 buffer^34^. This solution can usually be kept for several weeks without significant loss of ascorbate due to the lack of oxygen. Cells were treated with ascorbate for 1 h at 37 °C. Then the ascorbate-containing medium was removed, and cells are ready for the next phase of experimentation.

### Clonogenic survival assay

MIA PaCa-2 and ρ^0^ cells were seeded into 60 mm^2^ tissue culture dishes at 1.0 × 10^5^ to 1.5 × 10^5^ cells/dish in 4.0 mL of medium. Cells were cultured in their respective medium before exposure to P-AscH^-^ (2 mM, 9 pmol cell^−1^, 1 h), H_2_O_2_ (20 µM for 1 h) or 2-deoxyglucose (20 mM for 1 h) in DMEM-10% FBS. We have found that the best approach to specify dose of P-AscH^-^ is as moles per cell rather than nominal concentration^22,35^. Ascorbate has been shown to readily oxidize in cell culture media to generate H_2_O_2_. However, media containing pyruvate shows less H_2_O_2_, as pyruvate can scavenge H_2_O_2_^20,21^. Because of this phenomenon, we treated all cell lines in pyruvate-free media. Following treatment, exposure medium was removed, cells were counted (to determine mol per cell of exposure as well as seeding density) and then seeded into 6-well plates in 4.0 mL of their respective medium. Plates were then incubated for 7–14 days at 37 °C, 5% CO_2_. Surviving colonies were fixed with 70% ethanol and stained with Coomassie Blue. Colonies were counted as a cluster of at least 50 cells. Plating efficiency and surviving fraction were calculated.

### Measurement of prooxidant levels

Prooxidant levels (presumably H_2_O_2_) were measured at various time intervals by labeling cells for 15 min at 37 °C using the oxidation sensitive [5-(and-6)-carboxy-2′,7′-dichlorodihydrofluorescein diacetate; C6827; ThermoFisher Scientific] and insensitive [C-369; 5-(and-6)-carboxy-2′,7′-dichlorofluorescein diacetate] fluorescent dyes dissolved in DMSO with flow cytometric analysis as described^42^. The oxidation insensitive probe was utilized to control for changes in uptake, ester cleavage, and efflux so that differences in fluorescence can definitively be attributed to changes in oxidation of the probe. Intracellular hydroperoxide production was estimated by adding 10 μM DCFDA to cells. After incubation at 37 ºC for 20 min, cells were harvested using a cell scraper and transferred to a collection tube along with the floating cells in the incubation buffer. DCFH fluorescence were measured by flow cytometry using Becton Dickinson LSR II at the Flow Cytometry Facility of University of Iowa.

### Measurement of cellular metabolic flux

A Seahorse XF96 Extracellular Flux Analyzer (Agilent-Seahorse) was used to conduct all metabolic experiments. Cells were seeded into Seahorse V3-PS XF 96-well plates at 20,000 cells per well in 200 μL growth media. After 48 h, the cells were washed with DMEM assay media that was bicarbonate and phenol red free, but supplemented with 5 mM HEPES, 10 mM D-glucose, 2 mM L-glutamine and 1 mM Na pyruvate, with the pH of the media adjusted to pH 7.40 at 37 ^o^ C before each experiment: allowing proton efflux rate (PER) calculations when a proper buffer factor for the system is used. Oxygen consumption rate (OCR) was then measured with the standard mitochondrial stress test protocol and report generator (Agilent-Seahorse). Oligomycin A (2.5 μM), FCCP (0.3 µM), rotenone (5 µM) and antimycin A (5 µM) were used: these concentrations were previously optimized in independent experiments. At the end of experiments, the number of cells/well was determined using a Moxi cell counter. OCR was normalized using an average cell number/well and expressed as amol O_2_ cell^−1^ s^−1^^36^.

### Glycolytic rate assay

The Glycolytic Rate Assay (Agilent-Seahorse) provides a non-invasive measure of glycolysis in live cells. This assay allows a more quantitative estimate of proton production from glycolysis than extracellular acidification rate (ECAR) alone. In this assay ECAR can be converted to estimates of proton efflux rates (PER) due to glycolysis: with validated stoichiometries to lactate and production of H^+^ by Agilent Seahorse. The glycolytic rate assay involves recording basal measurements of ECAR, followed by sequential injections of 0.5 µM each of rotenone/antimycin A and 50 mM 2-deoxyglucose (2-DG). This approach removes the contribution of mitochondrial CO_2_ to ECAR and allows for accurate measurement of glycolysis-linked proton efflux rate (glycoPER). Briefly, cells were seeded into a Seahorse V3-PS XF 96-well plate. After 48 h, the cells were washed with assay media and then experiments were run using Agilent Seahorse XF glycolytic rate assay protocol and report generator. During the experiment, both ECAR and OCR were measured and used in calculating GlycoPER, basal PER, and compensatory PER; results were normalized to cell number and expressed as amol H^+^ cell^−1^ s^−1^.

### ATP rate assay

The Agilent-Seahorse ATP rate assay quantifies the rate of ATP production from glycolysis and oxidative phosphorylation simultaneously in live cells. The assay employs a sequential injection of 2 µM of oligomycin and 0.5 µM each of rotenone/antimycin A. Determining the rate of proton production due to glycolysis (glycoPER) and oxygen consumption due to oxidative phosphorylation (mitoOCR) allows stoichiometric estimates of ATP production by glycolysis and Ox Phos in live cells. Cells were seeded into a Seahorse V3-PS XF 96-well plate. After 48 h, the cells were washed with assay medium, and run following Agilent Seahorse XF real-time ATP rate assay protocol and report generator. The rates of glycoATP, mitoATP, and total ATP production were measured and normalized to cell number and expressed as amol ATP cell^−1^ s^−1^.

### Transmission electron microscopy

MIA PaCa-2 cells and ρ^0^ cells grown on 18 mm glass cover slips in a 12-well plate, fixed in 2% formaldehyde plus 2.5% glutaraldehyde in 0.1 M cacodylate buffer, pH 7.4 overnight at 4 °C and then post fixed with 1% osmium tetroxide for 1 h. Following serial alcohol dehydration, the samples were embedded in Epon 12 (Ted Pella, Redding, CA). Ultramicrotomy was performed, and ultrathin Sects. (70 nm) were post stained with uranyl acetate and lead citrate. Samples were visualized with a JEOL 1230 transmission electron microscope (Tokyo, Japan) at the Central Microscopy Research Facility of The University of Iowa.

### NAD^+^/NADH assay

NAD^+^/NADH levels were measured by the NAD/NADH-Glo Assay (Promega G9071). Cells were seeded in 60 mm dishes for 48 h. Cells were then treated with P-AscH^-^ (2 mM, 7 pmol cell^-1^) for 1 h in DMEM-10% FBS. After treatment, levels of NAD^+^ and NADH in samples were measured according to the manufacturer’s protocol; 12,500 cells were used per measurement for either NAD^+^ or NADH. The amounts of NAD^+^ and NADH in samples were calculated from the corresponding standard curve and then further transformed to an average intracellular concentration by using the cell volume, as measured with a Moxi Z Cell Counter.

### Antioxidant activity

3 × 10^6^ cells grown on 100-mm tissues culture dishes were detached by 0.25% trypsin–EDTA and the cell pellets were stored at -80 °C. For an assay, 70 µL of phosphate buffer (50 mM, pH 7.0) were added to the cell pellet and sonicated for 30 s using a sonicator. After sonication samples were centrifuged for 5 min at 10,000 g. Phosphate buffer (50 mM, pH 7.0, 500 µL) was added to a 10 mm pathlength quartz cuvette, followed by 20 µL of cell lysate and 250 µL of 30 mM H_2_O_2_. Catalase activity was measured in a HP UV–Vis Diode array Spectrophotometer. The absorbance at 240 nm was followed for 120 s with a cycle time of 10 s. The activity of catalase in cells was determined as m*k* Units per mg protein^37–39^.

GPx1 activity was measured by the method of Stolwijk et al.^40^. Cell pellets were resuspended in 200 µL GPx assay buffer (contains 100 mM Tris base, pH 8.0, 1.5 mM NaN_3_, 2.0 mM EDTA and 0.1% Triton X-100). After centrifugation at 10 000 g for 10 min, 50 µL supernatant were added to 1 mL quartz cuvette, followed by 50 µL each of 4 mM NADPH, 30 U mL^−1^ glutathione disulfide reductase and 60 mM glutathione, and 785 µL assay buffer. The assay mixture was incubated at 37 °C for 5 min. The loss of NADPH absorbance at 340 nm was then followed for about 300 s after addition of 15 µL of H_2_O_2_. The rate of NADPH oxidation (slope) for background, and with H_2_O_2_ were determined. The extinction coefficient ε_340_ = 6270 M^−1^ cm^−1^ at 37 °C and pH 8 was used to calculate the activity of GPx1. GPx1 activity was expressed as mU mg^−1^ protein.

### Intracellular ATP assay

Cells were cultured in 60 mm dishes for 48 h before treatment with P-AscH^-^ (2 mM, 7 pmol cell^-1^) for 1 h in DMEM-10% FBS. After treatment, cells were trypsinized and resuspended with PBS. Intracellular ATP was then determined with CellTiter Glo Luminescent Cell Viability Assay (Promega, cat. no. G7570) as described previously^6^. Data were transformed to an average intracellular concentration by using the cell volume, as measured with a Moxi Z Cell Counter.

### Statistical analysis

One-way ANOVA followed by Tukey’s post hoc was used to examine statistical differences between means for multiple comparisons. All means were calculated from at least three independent experiments, with error bars representing SEM. Test of statistical significance were performed using GraphPad Prism 7.0d. Western blot analyses were performed with at least two biological replicates.

## Supplementary Information

Supplementary Information.

## Data Availability

All the data described are contained within the manuscript and associated Supporting Information.
