# Supplementary material for: The role of mitochondria in pharmacological ascorbate-induced toxicity
Source: Sci Rep. 2022 Dec 29;12:22521. doi: 10.1038/s41598-022-27185-9 (PMC9800562; doi:10.1038/s41598-022-27185-9)
Supplement: Supplementary file 1 — Supplementary Information. [file 41598_2022_27185_MOESM1_ESM.docx]

**Supporting Information for**

**The Role of Mitochondria in**

**Pharmacological Ascorbate-induced Toxicity**

in

*Scientific Reports*

Juan Du^1^, Amanda N. Pope^1^, Brianne R. O’Leary^1^, Brett A. Wagner^2^, Prabhat C. Goswami^2^, Garry R. Buettner^2^, Joseph J. Cullen^1,2^

From the Department of Surgery^1^, and Free Radical and Radiation Biology Program, Department of Radiation Oncology^2^, The University of Iowa Carver College of Medicine, Iowa City, Iowa, USA.

November 2022

**Supporting Information Figure S1.**

**A**

**mtDNA**

**GAPDH
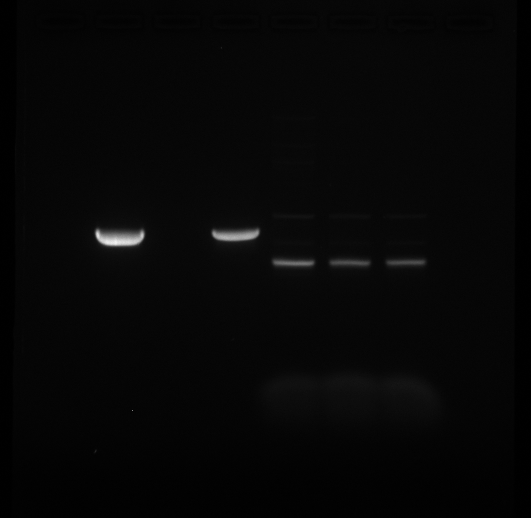
**

**GAPDH**

**mtDNA**

**B**

**Supporting Information Figure S1. Examples of clones with retained mtDNA.**

**A.** PCR analysis demonstrating retention of mtDNA in the 3790 clone 6.

**B.** Although much less than MIA PaCa-2 cells, the 3790 clone 6 maintained basal OCR. 3790 clone 6 also had typical responses to the mitochondrial stress test, as expected if mitochondrial function is retained.

**Supporting Information Figure S2.**

**Supporting Information Figure S2. Proton efflux rates (PERs) and ATP production rates of two *ρ*^0^ clones.**

1. PER of 3790 clone 5 and 4008 clone 3 (means ± SEM). Note the different scales for the ordinates. The 4008 clone 3 is producing ATP at only about 50% of the rate of the clone 3790 clone 5.
2. In the *ρ*^0^ clones 3790 clone 5 and 4008 clone 3, there is no production of ATP from mitochondria.

**Supporting Information Figure S3.**


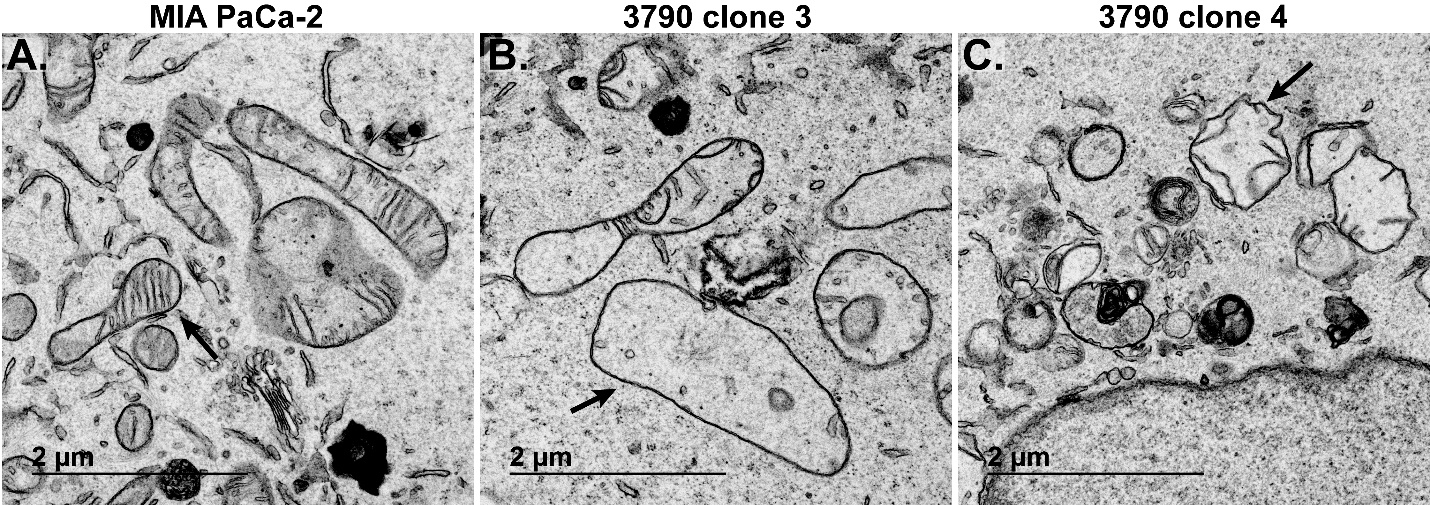


**Supporting Information Figure S3. Mitochondrial morphology changes in *ρ*^0^ clones when observed by transmission electron microscopy.**

**A.** MIA PaCa-2 cells show regularly arranged cristae (arrow) in intact mitochondria.

**B.** *ρ*^0^ 3790 clone 3 has enlarged mitochondria with near complete loss of the internal cristae structural pattern (arrow).

**C.** *ρ*^0^ 3790 clone 4 demonstrating loss of the cristae and irregular cristae structural pattern (arrow).

**Supporting Information Figure S4.**


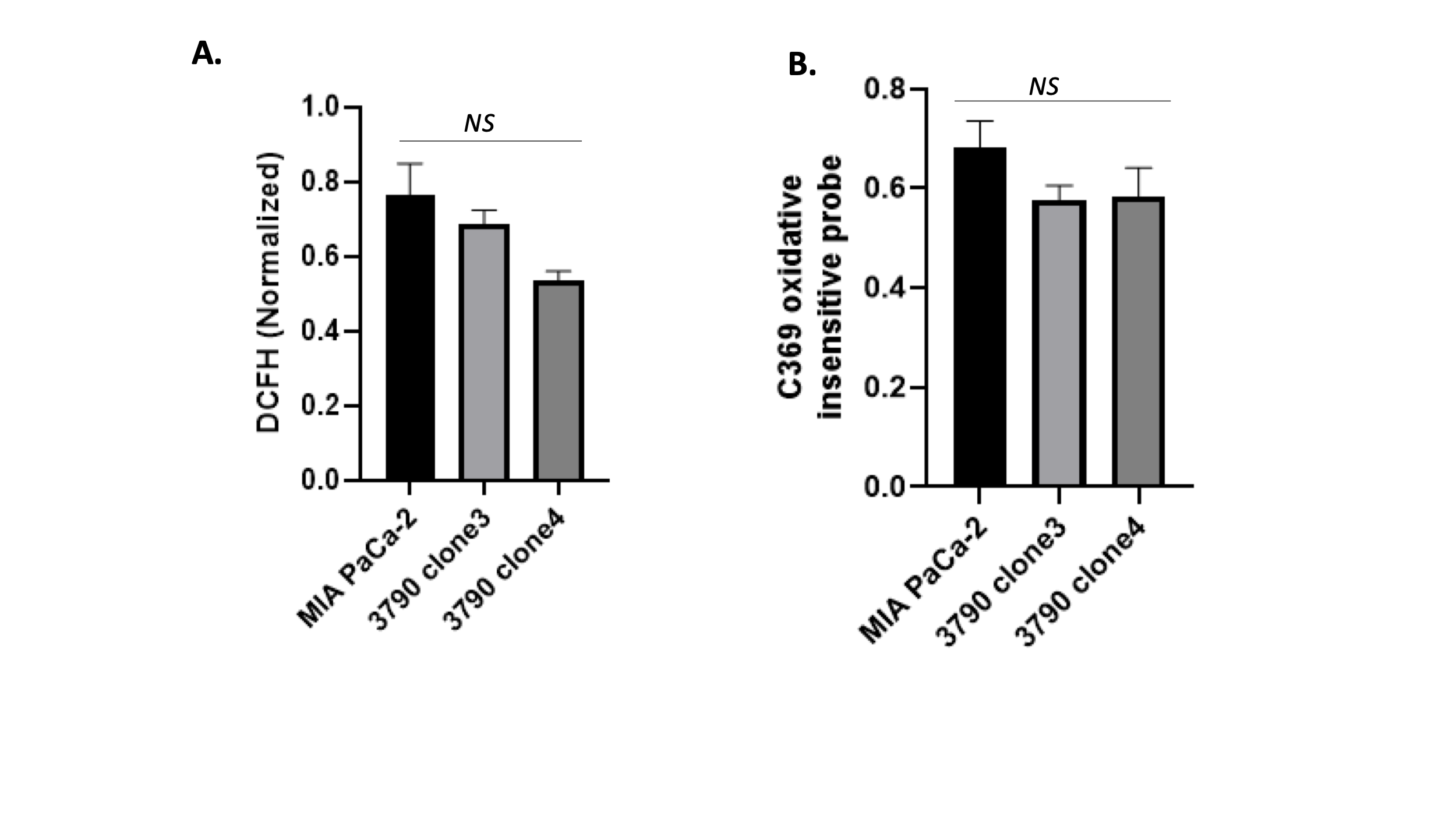


**Supporting Information Figure S4. No changes in DCF-DA staining in cell lines after P-AscH^-^.** MIA PaCa-2 and *ρ*^0^ clones were exposed to P-AscH^-^ 10 pmol/cell for 1 h in DMEM-10% FBS, then the cells were grown in their growth media for 4 h. DCF-DA fluorescence were measured by flow cytometry afterwards. One way ANOVA Tukey’s multiple comparison.

**A**. DCFH staining using the oxidation sensitive probe.

**B**. DCFH staining using C369 oxidation insensitive probe.

**Supporting Materials**

Catalase antibody (cat. no.14097), GPx1 antibody (cat. no. 3286), SOD1 antibody (cat. no. 37385), SOD2 (cat. no. 13194), PARP1 (cat. no. 9542), β-actin (cat. no. 4970), and α-tubulin (cat. no. 3873) were from Cell Signaling Technology. GAPDH (MAB374, Millipore), cytochrome c oxidase subunit 2 (COX2) antibody (sc-514489, Santa Cruz Biotechnology), γ- H2AX (phospho S139) (ab11174, abcam). Horseradish peroxidase-conjugated goat anti-rabbit (cat. no. AP307P) and goat anti-mouse (cat. no. AP308P) were from Millipore Sigma. CM-H_2_DCFDA (General Oxidative Stress Indicator) (Thermo Fisher Scientific, cat. no. C6827), and Carboxy-DCFDA (5-(and-6)-Carboxy-2',7'-Dichlorofluorescein Diacetate), mixed isomers from ThermoFisher Scientific (cat. no. C369).

All chemicals were from Sigma Aldrich except when specifically indicated.

**Supporting Information Figure S5.**

A
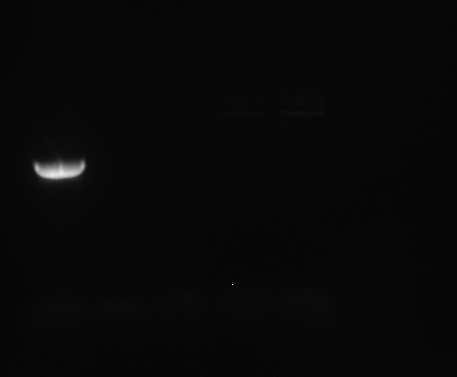


mtDNA 630 bp

B
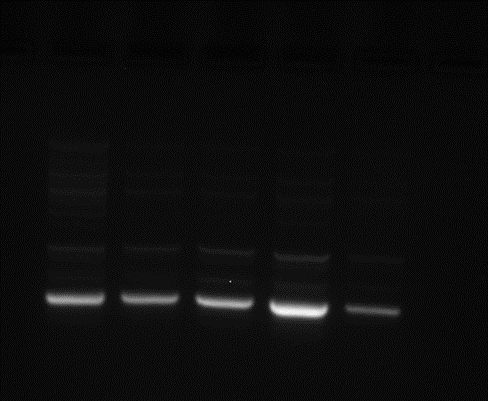


GAPDH

C
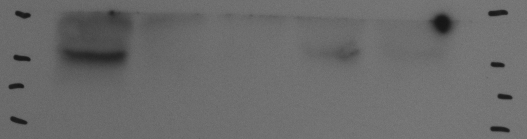


21 kDa

a

D
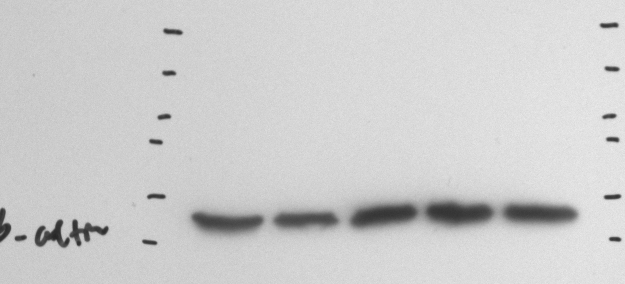


45 kDa

a

**Supporting Information Figure S5.** Original unprocessed DNA gel from main **Figure 1A** and unprocessed Western blots from main **Figure 1B**.

**A.** The 630 bp PCR product of MIA PaCa-2 and four ρ^0^ clones with short (left) and long exposure (right).

**B.** Corresponding GAPDH loading control from MIA PaCa-2 cells and four ρ^0^ clones.

**C.** mtCOX2 levels in MIA PaCa-2, 3790 clone 3, clone 4, clone 5 and 4008 clone 3.

**D.** Corresponding actin loading control from MIA PaCa-2 cells and four ρ^0^ clones.

**Supporting Information Figure S6**

A
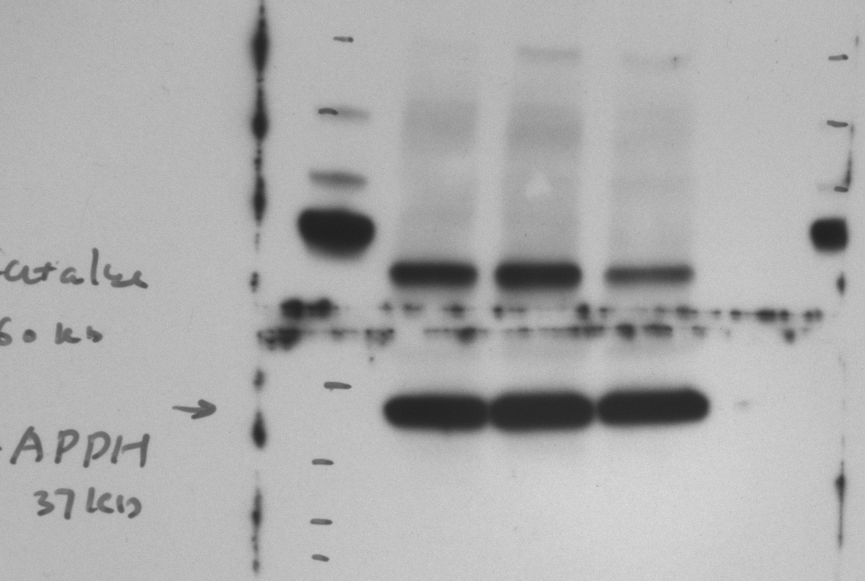


**60 kDa**

B
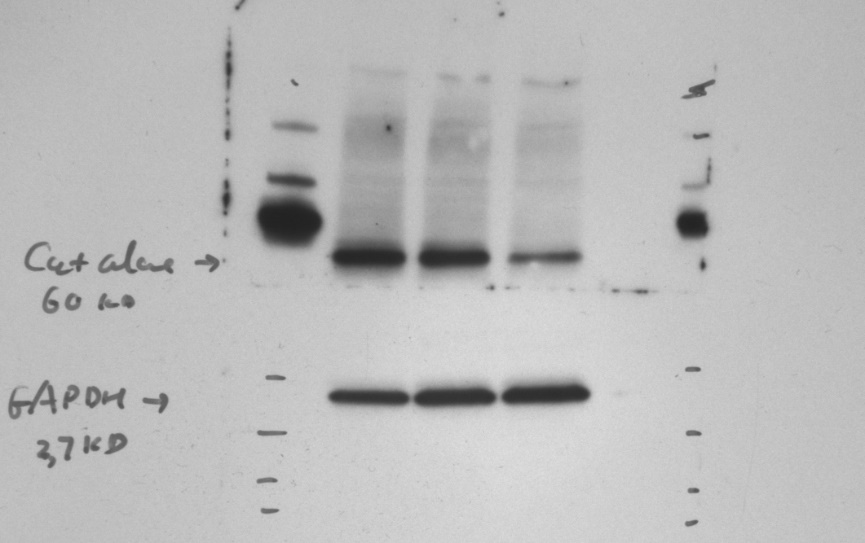


**37 kDa**

C
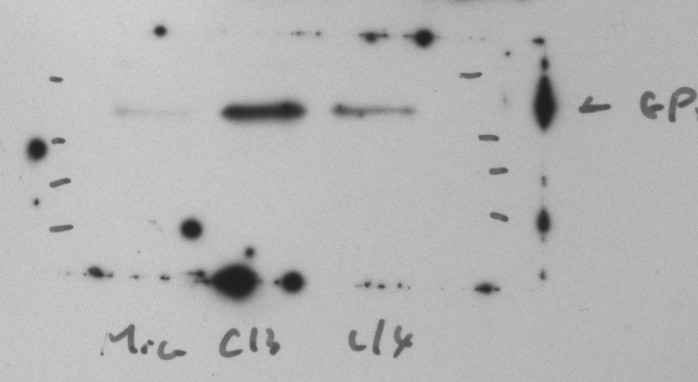


**22 kDa**

D
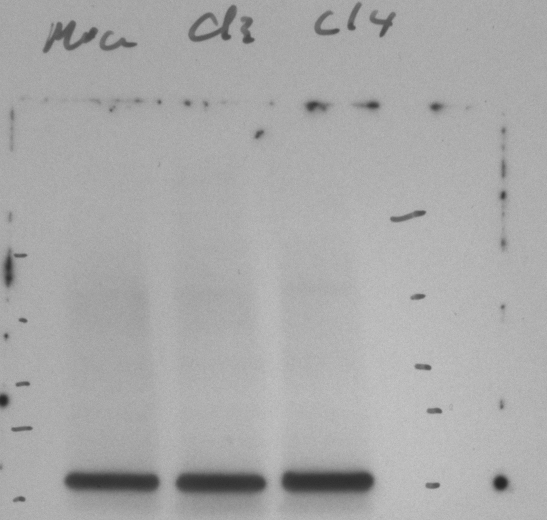


**55 kDa**

E
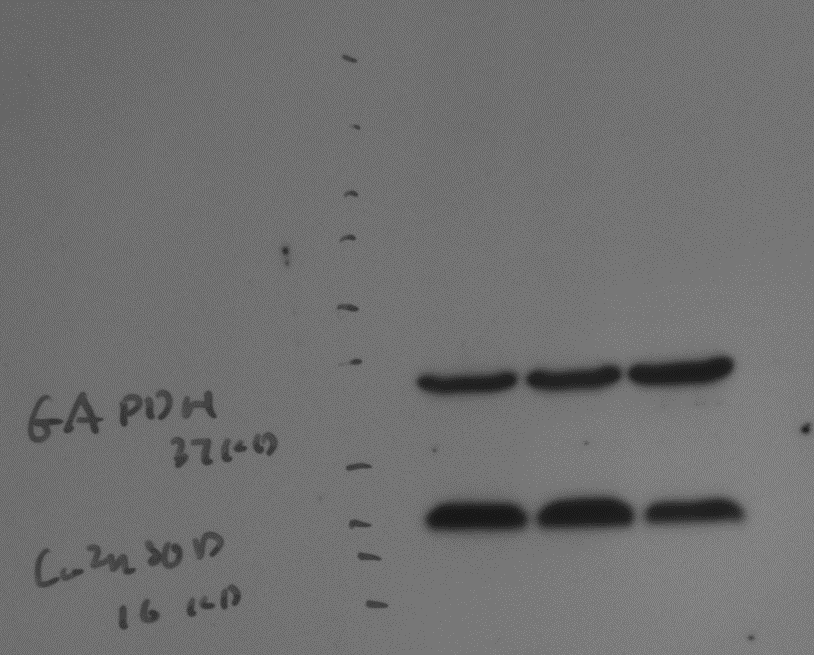


**16 kDa**

**37 kDa**

F
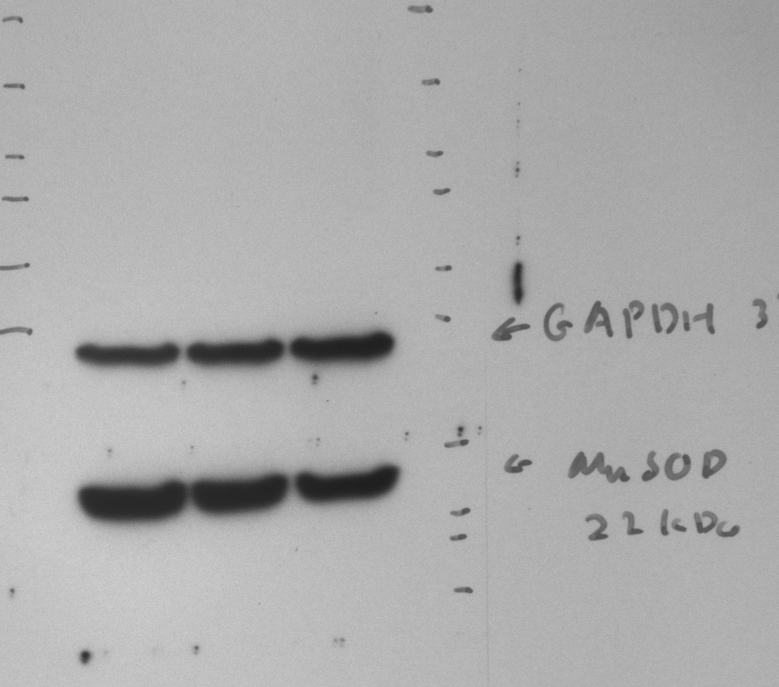


**22 kDa**

**37 kDa**

**Supporting Information Figure S6**. Original unprocessed Western blots from main **Figure 3C**.

**A.** Catalase levels in MIA PaCa-2, 3790 clone 3 and 3790 clone 4.

**B.** Corresponding GAPDH loading control from MIA PaCa-2 cells, 3790 clone 3 and 3790 clone 4.

**C.** GPx1 levels in MIA PaCa-2, 3790 clone 3 and 3790 clone 4.

**D.** Corresponding tubulin loading control from 3790 clone 3 and 3790 clone 4.

**E.** CuZnSOD levels in MIA PaCa-2 cells, 3790 clone 3 and 3790 clone 4 and corresponding GAPDH loading control.

**F.** MnSOD levels in MIA PaCa-2 cells, 3790 clone 3 and 3790 clone 4 and corresponding GAPDH loading control.

**Supporting Information Figure S7.**

A
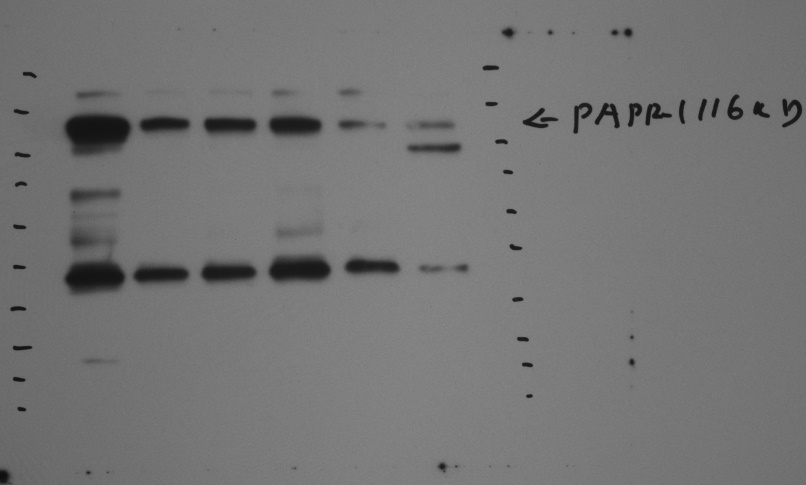


**116 kDa**

B
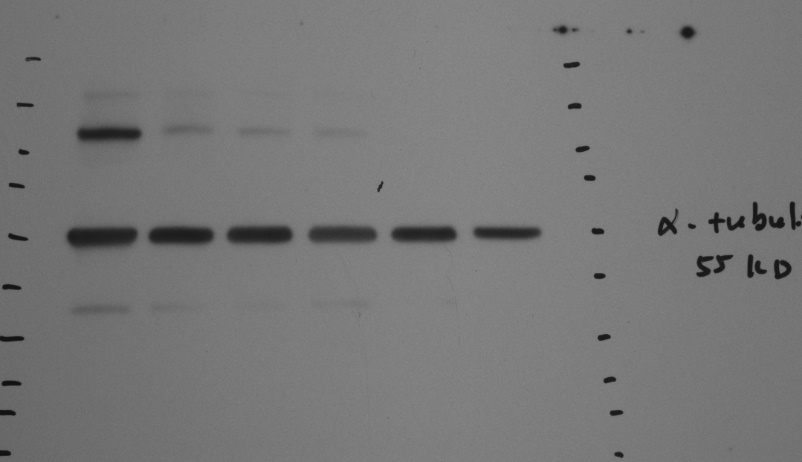


**55 kDa**

C
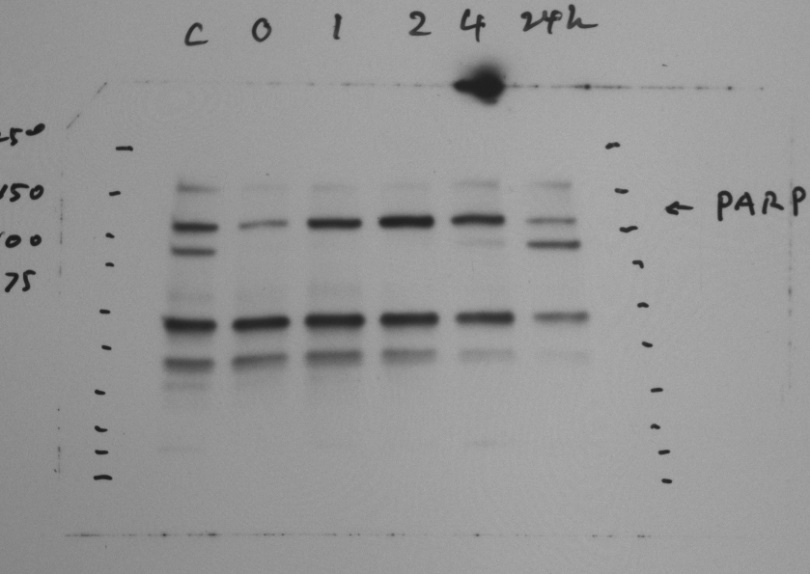


**116 kDa**

D
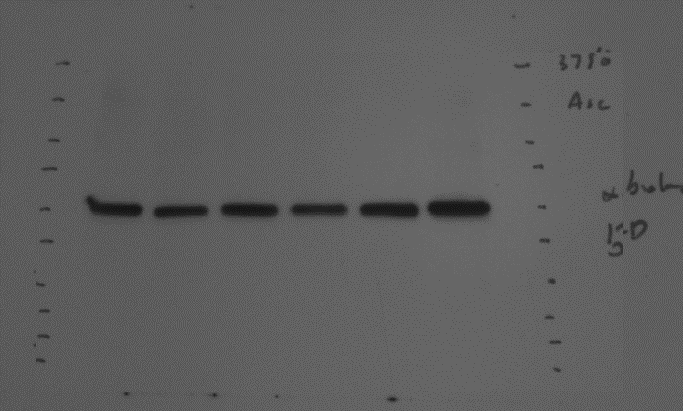


**55 kDa**

E
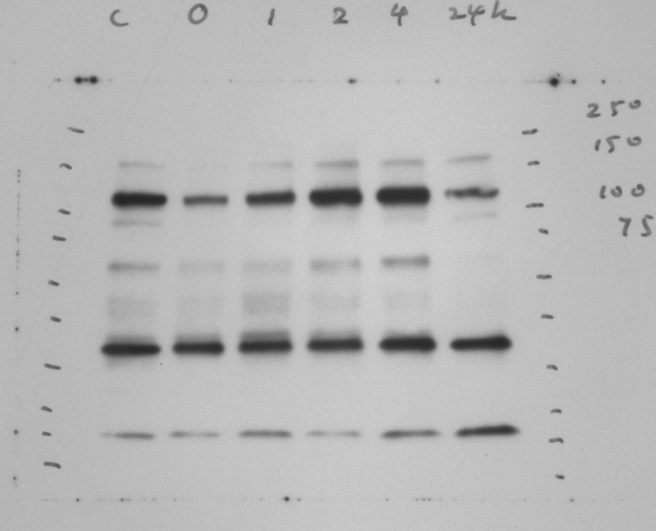


**116 kDa**

F
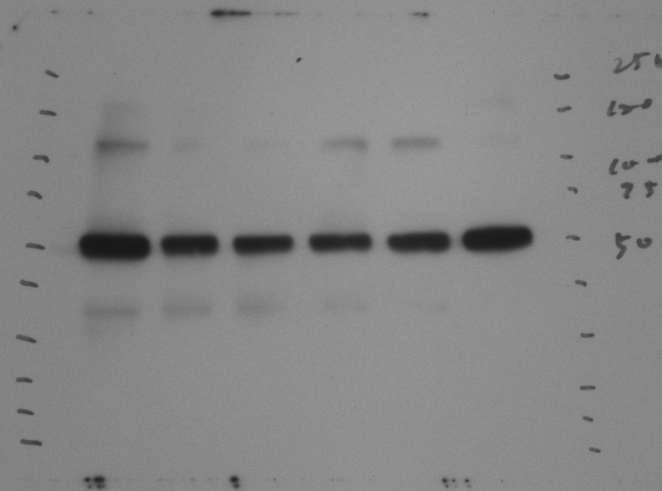


55 kDa

**Supporting Information Figure S7.** Original unprocessed Western blots from main **Figure 5A**.

**A.** PARP1 level in MIA PaCa-2 treated with P-AscH**^-^**.

**B.** Corresponding tubulin loading control from MIA PaCa-2 cells.

**C.** PARP1 level in 3790 clone 3 treated with P-AscH**^-^**.

**D.** Corresponding tubulin loading control from 3790 clone 3.

**E.** PARP1 level in 3790 clone 4.

**F.** Corresponding tubulin loading control from 3790 clone 4.

**Supporting Information Figure S8**

A **
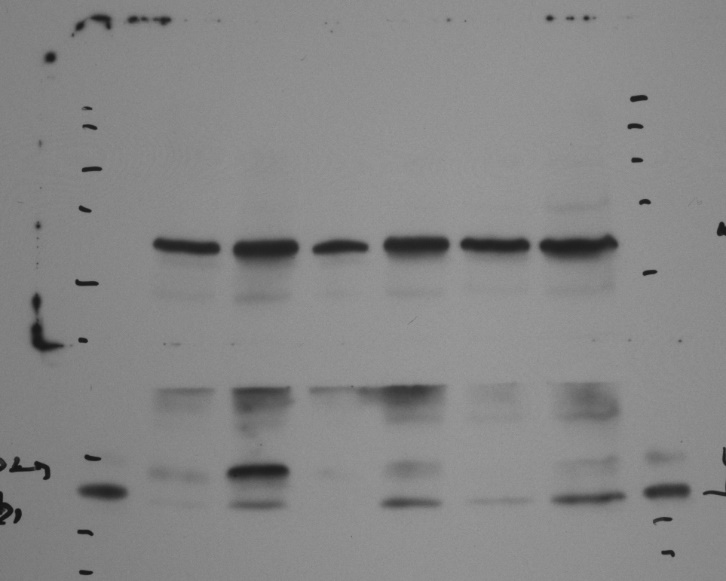
**

**22 kDa**

**17 kDa**

**55 kDa**

**Supporting Information Figure S8.** Original unprocessed Western blots from main **Figure 5B**.

**A**. γH2AX and γH2AX-ubiquitinated level in MIA PaCa-2, 3790 clone 4, and 3790 clone 3 control and P-AscH**^-^** treated cells.

End Supporting Information
